# Research on the construction of a knowledge graph for tomato leaf pests and diseases based on the named entity recognition model

**DOI:** 10.3389/fpls.2024.1482275

**Published:** 2024-11-07

**Authors:** Kun Wang, Yuyuan Miao, Xu Wang, Yuze Li, Fuzhong Li, Haiyan Song

**Affiliations:** ^1^ Software College, Shanxi Agricultural University, Jinzhong, Shanxi, China; ^2^ Agricultural Engineering College, Shanxi Agricultural University, Jinzhong, Shanxi, China

**Keywords:** tomato leaf pests and diseases, knowledge graph, ALBERT-BiLSTM-CRF, Neo4j graph database, digital diagnosis

## Abstract

**Introduction:**

Tomato leaf pests and diseases pose a significant threat to the yield and quality of Q6 tomatoes, highlighting the necessity for comprehensive studies on effective control methods.

**Methods:**

Current control measures predominantly rely on experience and manual observation, hindering the integration of multi-source data. To address this, we integrated information resources related to tomato leaf pests and diseases from agricultural standards documents, knowledge websites, and relevant literature. Guided by domain experts, we preprocessed this data to construct a sample set.

**Results:**

We utilized the Named Entity Recognition (NER) model ALBERT-BiLSTM-CRF to conduct end-to-end knowledge extraction experiments, which outperformed traditional models such as 1DCNN-CRF and BiLSTM-CRF, achieving a recall rate of 95.03%. The extracted knowledge was then stored in the Neo4j graph database, effectively visualizing the internal structure of the knowledge graph.

**Discussion:**

We developed a digital diagnostic system for tomato leaf pests and diseases based on the knowledge graph, enabling graphical management and visualization of pest and disease knowledge. The constructed knowledge graph offers insights for controlling tomato leaf pests and diseases and provides new research directions for pest control in other crops.

## 1 Introduction

Agricultural production plays a crucial role in social development. In practical agricultural activities, crops are easily affected by various adverse factors. Among them, pests and diseases are one of the most significant factors affecting crop yield and income (Abdullah et al., 2023). Crop diseases and insect pests are characterized by their wide spread and propensity to cause disasters, which seriously affects the healthy growth of crops. Tomato, as a widely cultivated high-value vegetable globally, inevitably faces various factors such as improper cultivation practices, inadequate control measures, and environmental pollution during its cultivation process. This results in the occurrence of seventeen common types of pests and diseases, such as damping-off, viral diseases and leaf mold et al. These diseases not only severely impact the quality of tomatoes, but also significantly reduce yields, leading to substantial economic losses.

The traditional relational database knowledge management methods are unable to effectively represent and store such knowledge, facing challenges such as difficulties in integrating heterogeneous data, inefficiencies in expressing data relationships, and inadequacies in knowledge refinement. The term “knowledge graph” was used by Edgar W. Schneider in 1972 to describe data structures and control flows in educational modules (Schneider, 1973). In 2012, Google introduced the Google Knowledge Graph, effectively expressing relationships between data, marking the formal naming of knowledge graphs (Singhal, 2012). Research and construction of Knowledge Graphs have penetrated various domains of social life such as education (Xue et al., 2024), healthcare systems (Shi et al., 2017; Wu et al., 2023), and smart cities (Liu et al., 2021). They represent entities, attributes, and their relationships from the natural world in a graphical form, allowing for a better expression of domain-specific information closer to human cognitive understanding (Wu et al., 2021a). Based on the scope of knowledge coverage, Knowledge Graphs are mainly classified into two types: vertical Knowledge Graphs and general Knowledge Graphs (Ji et al., 2021). General Knowledge Graphs cover a wide range of knowledge across multiple domains, primarily addressing common-sense issues in the real world. Currently influential general Knowledge Graphs include YAGO2 (Hoffart et al., 2011), DBpedia (Lehmann et al., 2015), Freebase (Bollacker et al., 2008), as well as Chinese Knowledge Graph websites such as CN-DBpedia (Xu et al., 2017) and Baidu Zhixin (Niu et al., 2011). General Knowledge Graphs can be likened to “structured encyclopedic knowledge bases,” emphasizing breadth of knowledge and commonly used in search engines, constructed mainly in a bottom-up manner. Vertical Knowledge Graphs, also known as domain-specific Knowledge Graphs, are typically tailored to specific domains and require higher levels of depth and accuracy in knowledge representation. They serve as specialized knowledge repositories based on industry needs. For instance, Xindong Wu and colleagues proposed the Huapu-KG model, integrating HAO intelligence (human intelligence + artificial intelligence + organizational intelligence) for constructing genealogy Knowledge Graphs, addressing challenges of heterogeneity, autonomy, complexity, and evolution in genealogy data (Wu et al., 2021b). Wang, Chengbin et al. utilized hybrid corpora, Chinese word segmentation rules, and statistical methods to build a Knowledge Graph, demonstrating the potential and practicality of natural language processing and Knowledge Graph technologies in Earth science research (Wang et al., 2018). Yu, Tong et al. integrated traditional Chinese medicine terms, documents, and databases into a large-scale Knowledge Graph for visualization, retrieval, and recommendation services, facilitating the sharing, interpretation, and utilization of knowledge in traditional Chinese medicine and health preservation. Furthermore, some researchers combine Knowledge Graphs with specific technologies to address particular issues (Yu et al., 2017). For example, Zhuotong Li et al. proposed a method using Knowledge Graphs and graph neural networks for fault localization, demonstrating excellent accuracy in analyzing network anomalies on the SDON platform, thereby supporting industrial-scale alarm analysis and fault localization (Li et al., 2021). Qi Chen et al. introduced a novel zero-shot learning method using Knowledge Graphs to classify a large amount of social text data, achieving superior performance over six state-of-the-art NLP deep learning models on COVID-19-related tweet datasets (Chen et al., 2021). This method resolves issues with deep learning models requiring extensive labeled data and enhances the processing and analysis capabilities of CPSS data. Yi Luo et al. proposed the DTKGIN model, combining Knowledge Graphs and intent graphs to predict drug-target interactions, effectively addressing issues of high sparsity and cold start, demonstrating its effectiveness in predicting potential drug-target interactions through case studies (Luo et al., 2024).

Traditional relational database knowledge management methods cannot effectively represent and store such knowledge, facing issues such as inability to integrate heterogeneous data, inefficiency in expressing data relationships, and inability to refine knowledge. Knowledge Graph is a method proposed by Google in 2012 to effectively express relationships between data through a semantic network (Singhal, 2012). Research and construction of Knowledge Graphs have penetrated various domains of social life such as education (Xue et al., 2024), healthcare systems (Shi et al., 2017; Wu et al., 2023), and smart cities (Liu et al., 2021). They represent entities, attributes, and their relationships from the natural world in a graphical form, allowing for a better expression of domain-specific information closer to human cognitive understanding (Wu et al., 2021a).Based on the scope of knowledge coverage, Knowledge Graphs are mainly classified into two types: vertical Knowledge Graphs and general Knowledge Graphs (Ji et al., 2021). General Knowledge Graphs cover a wide range of knowledge across multiple domains, primarily addressing common-sense issues in the real world. Currently influential general Knowledge Graphs include YAGO2 (Hoffart et al., 2011), DBpedia (Lehmann et al., 2015), Freebase (Bollacker et al., 2008), as well as Chinese Knowledge Graph websites such as CN-DBpedia (Xu et al., 2017) and Baidu Zhixin (Niu et al., 2011). General Knowledge Graphs can be likened to “structured encyclopedic knowledge bases,” emphasizing breadth of knowledge and commonly used in search engines, constructed mainly in a bottom-up manner. Vertical Knowledge Graphs, also known as domain-specific Knowledge Graphs, are typically tailored to specific domains and require higher levels of depth and accuracy in knowledge representation. They serve as specialized knowledge repositories based on industry needs. For instance, Xindong Wu and colleagues proposed the Huapu-KG model, integrating HAO intelligence (human intelligence + artificial intelligence + organizational intelligence) for constructing genealogy Knowledge Graphs, addressing challenges of heterogeneity, autonomy, complexity, and evolution in genealogy data (Wu et al., 2021b). Wang, Chengbin et al. utilized hybrid corpora, Chinese word segmentation rules, and statistical methods to build a Knowledge Graph, demonstrating the potential and practicality of natural language processing and Knowledge Graph technologies in Earth science research (Wang et al., 2018). Yu, Tong et al. integrated traditional Chinese medicine terms, documents, and databases into a large-scale Knowledge Graph for visualization, retrieval, and recommendation services, facilitating the sharing, interpretation, and utilization of knowledge in traditional Chinese medicine and health preservation. Furthermore, some researchers combine Knowledge Graphs with specific technologies to address particular issues (Yu et al., 2017). For example, Zhuotong Li et al. proposed a method using Knowledge Graphs and graph neural networks for fault localization, demonstrating excellent accuracy in analyzing network anomalies on the SDON platform, thereby supporting industrial-scale alarm analysis and fault localization (Li et al., 2021). Qi Chen et al. introduced a novel zero-shot learning method using Knowledge Graphs to classify a large amount of social text data, achieving superior performance over six state-of-the-art NLP deep learning models on COVID-19-related tweet datasets (Chen et al., 2021). This method resolves issues with deep learning models requiring extensive labeled data and enhances the processing and analysis capabilities of CPSS data. Yi Luo et al. proposed the DTKGIN model, combining Knowledge Graphs and intent graphs to predict drug-target interactions, effectively addressing issues of high sparsity and cold start, demonstrating its effectiveness in predicting potential drug-target interactions through case studies (Luo et al., 2024).

Building a Knowledge Graph primarily involves four steps: data acquisition, knowledge extraction, knowledge fusion, and knowledge processing. Knowledge extraction, as a core step in the construction of a knowledge graph, is responsible for accurately obtaining useful knowledge from vast and complex data related to crop pests and diseases. This includes extracting information on symptoms, control measures, disease categories, distribution ranges, causes of disease, and locations of occurrence. Traditional methods of knowledge extraction typically rely on manually defined rules or statistical models, which excel in adaptability to specific domain languages and rules. However, they face limitations in performance and generalization capability when dealing with the complex contexts and diverse textual data of the current information explosion era. Deep learning effectively addresses many complex problems that traditional methods struggle with. Deep learning-based knowledge extraction significantly enhances the efficiency and accuracy of information processing. It automates the learning and extraction of abstract features from data, eliminating the need for manually designed feature engineering and greatly simplifying the data processing workflow. Moreover, deep learning models leverage robust language models and sequence modeling capabilities to better understand the grammar structure, semantic relationships, and contextual information within texts. Currently, many scholars extensively explore and apply deep learning models in the knowledge extraction process. For example, Bihui Yu et al. proposed a new entity recognition model based on character embedding, Iterated Dilated Convolutional Neural Networks(IDCNN), and Conditional Random Fields(CRF), demonstrating excellent performance in corpus experiments in the military equipment domain (Yu and Wei, 2020). Lample et al. introduced the Bi-LSTM-CRF model, which achieved high entity extraction evaluation metrics by incorporating a CRF module on top of a BiLSTM network in corpus datasets (Lample et al., 2016). In 2018, Luo et al. enhanced the Bi-LSTM-CRF model with an Attention mechanism, achieving an F1 score of over 90% on public document-level datasets (Luo et al., 2018). In the same year, Devlin et al. introduced the BERT pre-training model, generating deep bidirectional semantic representations based on input corpus to fully represent contextual information, thereby aiding multiple natural language processing tasks (Devlin et al., 2019). BERT’s advent brought significant breakthroughs to entity recognition technology. In 2020, Google proposed the ALBERT model (Lan et al., 2019), reducing computational complexity compared to BERT and ensuring stable model training. Xiao Zhang et al. tackled challenges in the terahertz domain’s QA system with long and short entity issues, proposing an entity recognition method based on ALBERT-BiLSTM-CRF (Zhang et al., 2020). They leveraged pre-trained deep bidirectional models to comprehensively understand sentence semantics, effectively improving the accuracy of long entity recognition.

After comparing existing entity recognition methods, we selected a fine-tuned ALBERT model with a BiLSTM-CRF layer as the final entity recognition model. We developed an efficient and accurate method for extracting knowledge about tomato leaf pests and diseases. The extracted triplet data was stored in a graph database, enabling the visualization and knowledge inference of the tomato leaf pest and disease knowledge graph. This allows complex agricultural knowledge to be clearly and intuitively presented to agricultural professionals. The main contributions of this study are as follows:

1. Addressing issues such as diverse data types, complex attributes and relationships, challenging deep-level association mining between data, and weak reasoning interpretability in traditional expert systems in tomato leaf disease and pest data. We utilized the ALBERT-BiLSTM-CRF model to extract knowledge about tomato diseases and pests, including symptoms, control measures, disease categories, distribution ranges, causes, and locations. By analyzing commonalities and characteristics among different diseases and pests, we organically linked information about various tomato diseases and pests, reducing redundant work and enhancing the efficiency and comprehensiveness of tomato disease and pest control.
2. To address the lack of open-source specialized corpora for tomato leaf disease and pest issues, we extracted various data about tomato diseases and pests from national or local standard documents. We employed web scraping techniques to gather data from professional agricultural knowledge websites and scanned texts from specialized books and literature on tomato leaf disease and pest control. With assistance from domain experts, we completed data preprocessing to form the domain-specific corpora required for constructing the tomato leaf disease and pest Knowledge Graph. The ALBERT-BiLSTM-CRF model was used to extract knowledge from sample data stored in a Neo4j database, establishing a rich, logically strong, and widely applicable Knowledge Base for tomato leaf disease and pest.
3. Based on the above solutions, we designed a digital diagnostic system for tomato leaf disease and pest integrated with advanced software development frameworks. This system combines deep learning algorithms and graph databases to process, analyze, and store information about tomato leaf disease and pest, providing users with precise prevention and control recommendations.

## 2 Materials and methods

### 2.1 Knowledge graph construction process

#### 2.1.1 Knowledge graph schema construction

A well-designed model layer facilitates the reuse of domain knowledge, creating a shared understanding of domain information and knowledge between humans and machines. A well-structured hierarchical ontology ensures the quality of knowledge extraction during the construction of the knowledge graph data layer, playing an irreplaceable role throughout the entire knowledge graph construction process (Guo et al., 2024). Referring to the ontology construction method from Stanford, we mainly followed six steps to construct the ontology for the knowledge graph in this study: determining the domain and scope, listing the main elements, defining the terms and the relationships between terms, defining classes and the class hierarchy, defining properties and relationship constraints, and instantiating the ontology.

The knowledge graph we constructed is focused on the agricultural domain, specifically on the ontology construction related to tomato leaf pests and diseases. We have tried to summarize as many elements as possible that should appear in the knowledge graph. **Table 1** lists the main elements included in the knowledge graph for the domain of tomato leaf pests and diseases.

**Table 1:** Some elements included in the knowledge graph for the domain of tomato leaf pests and diseases.

Subdomain Knowledge Graph	Main Elements
Tomato Leaf Diseases	Disease name, alias, symptoms, physical control, chemical control, biological control, disease category, distribution range, causes, affected parts, pathogen, etc.
Tomato Leaf Pests	Pest name, alias, phylum, class, order, family, genus, morphological characteristics, habits, symptoms, physical control, chemical control, biological control, occurrence area, affected parts, foreign name, etc.

**Figure 1:**
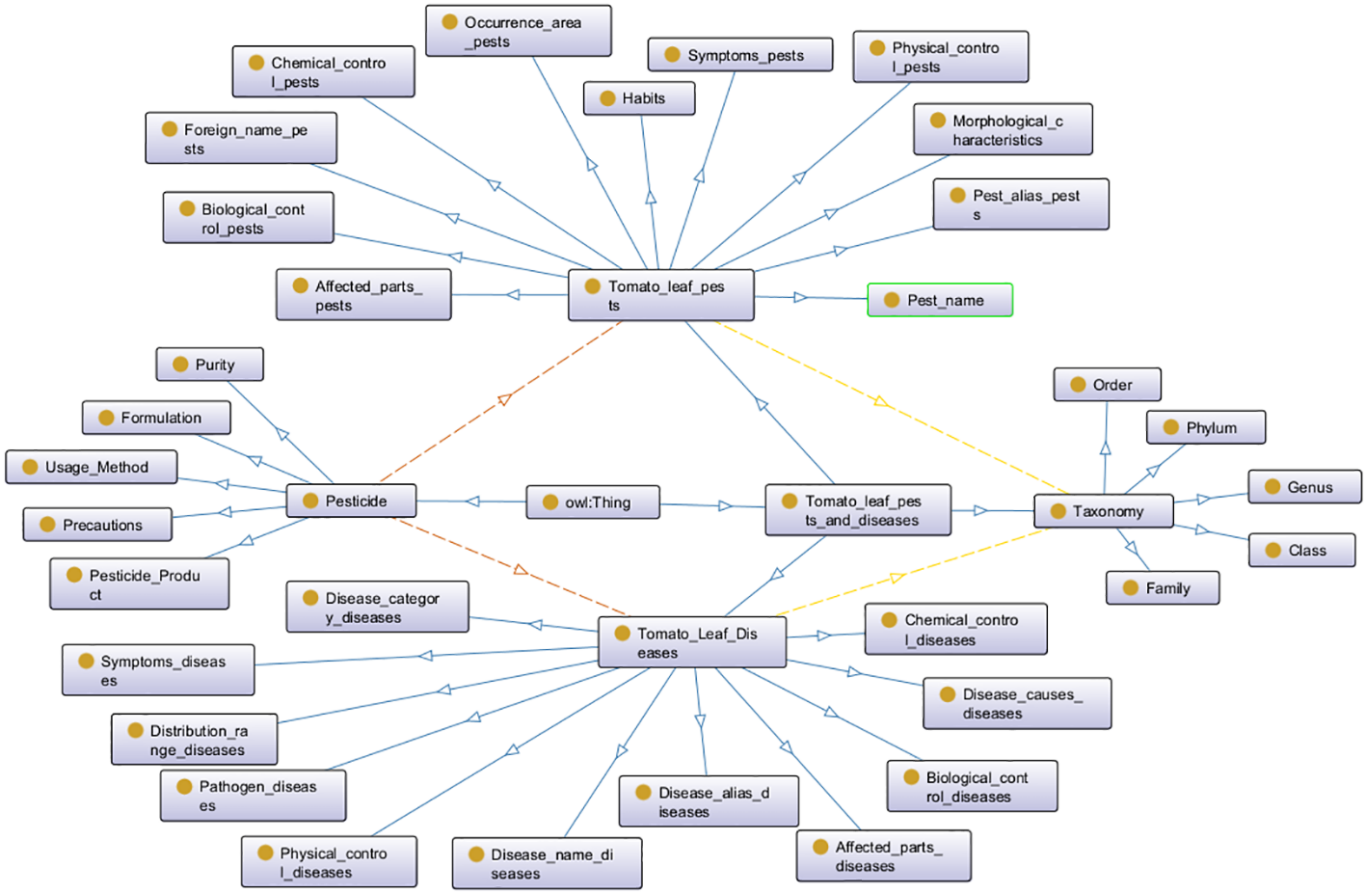
Ontology hierarchy diagram for the tomato leaf pests and diseases knowledge graph.

Next, we organize the domain-specific terms and the relationships and attributes between them. **Table 2** lists some professional terms and their relationships for two sub-domain knowledge graphs. Based on this, we define constraints and regulations for class relationships, intrinsic properties, and extrinsic properties. Finally, the defined properties, relationships, and constraints are used to instantiate the ontology. The hierarchical structure diagram of the tomato leaf pest and disease model layer, constructed using the Protégé tool, is shown in **Figure 1**.

**Table 2:** Sub-domain knowledge graph terms and relationships between terms.

Subdomain knowledge graph	Technical terms	Relationships
Tomato leaf diseases	Alias, Chinese name, Foreign name, Stem, Fruit, Flower, Ventilation and drainage, Proper spacing	Aliases, Affected parts, Harmful periods, Control methods, etc.
Tomato Leaf Pests	Alias, Leaves, Lepidoptera, Phylum Mollusca, Class Gastropoda, Order Stylommatophora, Family Limacidae, Genus Liriomyza Coleoptera, Natural enemies, Traps, Manual trapping	Aliases, Affected parts, Classification, Control methods, etc.

#### 2.1.2 Construction of the knowledge graph data layer

We used natural language processing methods and techniques to construct the data layer for tomato leaf pests and diseases. The sequence labeling strategy we selected is based on the BIO method, where B represents the beginning part of a tomato leaf pest or disease entity, I represents a non-beginning part of the entity, and O represents a non-entity, as shown in **Table 3**.

**Table 3:** BIO data annotation example.

Character	BIO annotation
powdery	B-DiseaseName
mildew	I-DiseaseName
mainly	O
affects	O
leaves	B-DiseaseLocation

The quality of data sample annotation determines the effectiveness of the model’s entity extraction. Considering that manual annotation is time-consuming, labor-intensive, and often lacks accuracy, we used the entity annotation software Colabeler to assist in annotating preprocessed data samples. During the annotation process, controversial areas were discussed with domain experts to ensure the accuracy of the results.

**Figure 2:**
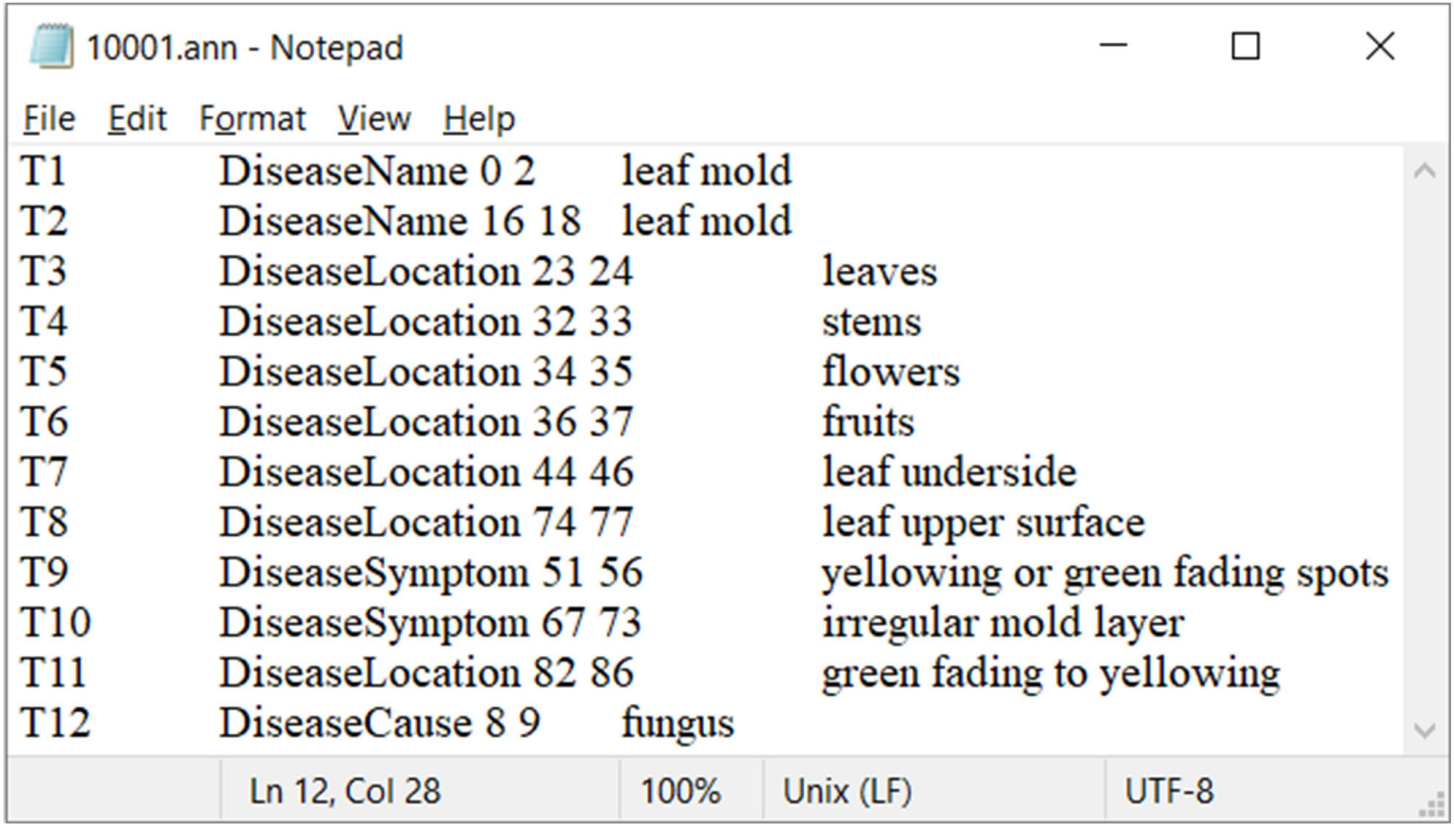
‘10001.ann’ file.

The annotated sample data is saved with files in the “.ann” format, as shown in **Figure 2**. In the “.ann” file, T1 represents the unique identifier or label for the annotation. “DiseaseName” indicates that the annotation marks a disease name in the text, with the number “0” representing the starting position of the annotation (i.e., starting from the 0th character), and the number “2” representing the ending position of the annotation (i.e., ending at the 2nd character). In the “10001.ann” file, T2 to T12 are similar to T1.

**Algorithm 1** outlines the process for generating BIO format data required for the NER task. The “Generate_BIO” algorithm traverses all annotated “.ann” files and corresponding “.txt” files in the specified directory, converting the data in each annotation file into BIO format (training data files).

Algorithm 1 **Generate_BIO.**

**Input:**”.ann” files and “.txt” files
**Output:**BIO Format Data
1 DEFINE root_dir、stream_path
2 file_list = glob.glob(root_dir + ‘/*.ann’)
3 FOR
4  ann_path = normalize_path(ann_path)
5  txt_path = convert_to_txt_path(ann_path)
6 .strip() Remove whitespace characters from the ends of text or label strings.
7  IF ann!= ‘ ‘
8   Assign the label “O” to all text.
9   FOR
10    TRY:
11     T, typ, word = split_tabs_and_strip(line)
12     t, s, e = typ.split()
13     s, e = convert_positions_to_int(s, e)
14     label[s] = concatenate(‘B-’, t)
15     while s< subtract(e, 1):
16     s = add(s, 1)
17     label[s] = concatenate(‘I-’, t)
18    EXCEPT:
19     continue
20    Connecting Text and BIO Labeling Using Spaces
21   END
22  print (ann_path, e)
23 END

### 2.2 ALBERT-BiLSTM-CRF Model

**Figure 3:**
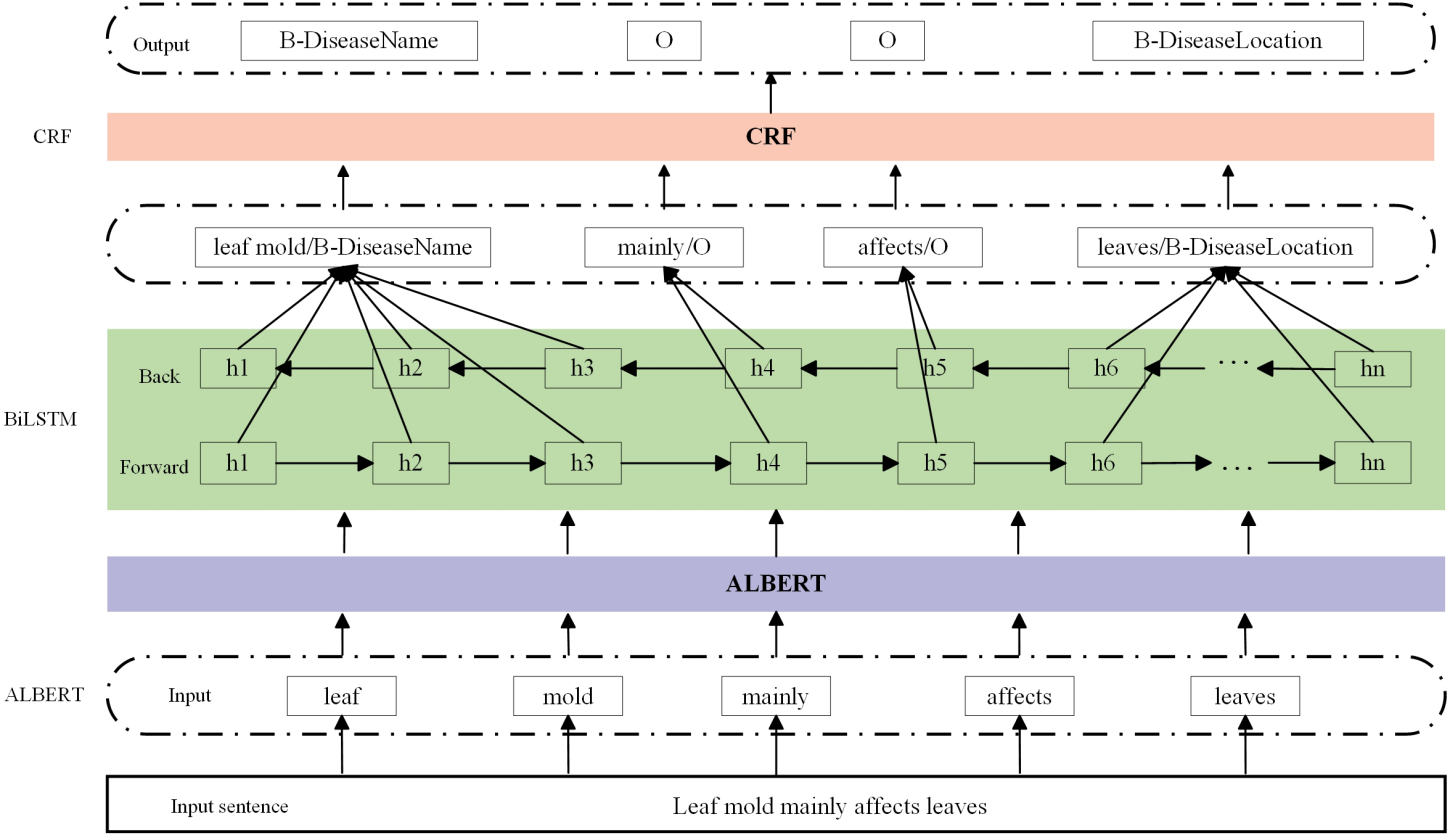
ALBERT BiLSTM CRF model structure.

We use the ALBERT-BiLSTM-CRF model to perform entity extraction tasks on tomato leaf pest and disease data samples. The model consists of three parts: ALBERT(A Lite Bidirectional Encoder Representations from Transformers), BiLSTM(Bi-directional Long Short-Term Memory), and CRF(Conditional Random Field), with the model structure illustrated in **Figure 3**. ALBERT is a pre-trained language model capable of understanding and processing natural language text; BiLSTM is a type of recurrent neural network designed for handling sequential data; and CRF is a conditional random field used to identify structural patterns in sequences. For example, in the sample “Leaf mold mainly affects leaves”, we use ALBERT as the pre-trained model to capture the semantic information of each word in the given context. First, the model tokenizes the sentence and converts it into word embeddings, including [‘*leaf mold*’, ‘*mainly’*, ‘ *affects’*, ‘*leaves’*]. ALBERT processes these word embeddings through its self-attention mechanism, considering the contextual information of each word in the sentence, and generates contextually relevant representations, such as [‘*leaf mold*_*emb’*, ‘*mainly_emb*’, ‘*affects_emb*’, ‘*leaves_emb*’]. These representations are then used as input to the BiLSTM model. BiLSTM models the sequence of each word’s contextual representation to capture the semantic information of words in the sentence. The output of the ALBERT layer, [‘*leaf mold*_*emb’*, ‘*mainly*_*emb’*, ‘*affects*_*emb’*, ‘*leaves*_*emb’*], is fed directly into the BiLSTM. The BiLSTM outputs a sequence of hidden states for each word, such as [‘*leaf mold*_*hidden’*, ‘*mainly*_*hidden’*, ‘*affects*_*hidden’*, ‘*leaves*_*hidden’*], which reflect each word’s semantic representation in its context. The BiLSTM output sequence is typically used as input to the CRF layer. The CRF layer combines the tag probability sequences output by the BiLSTM with a predefined transition matrix and uses the Viterbi algorithm to find the optimal tag sequence. For the entity recognition task of leaf mold, the optimal tag sequence might be [‘*B*-*DiseaseName’*, ‘*O*’, ‘*O*’, ‘*B-DiseaseLocation*’].

#### 2.2.1 ALBERT

**Figure 4:**
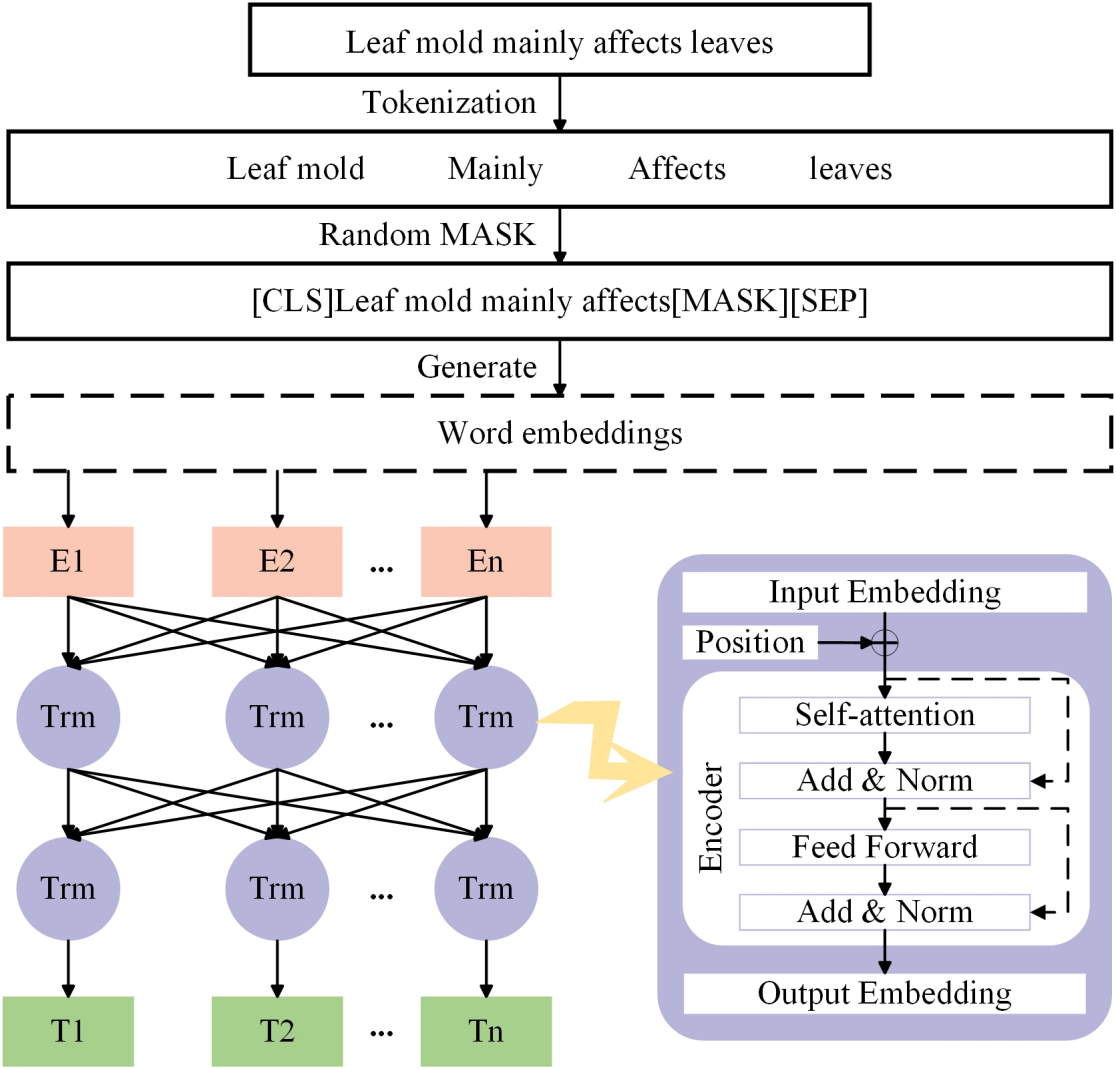
ALBERT model structure.

ALBERT, which stands for A Lite BERT, is a lightweight version of the BERT model. In the field of deep learning, the ALBERT model has gained significant attention for its outstanding performance (Lan et al., 2020). The ALBERT model consists of two parts: MASK preprocessing and a bidirectional Transformer, with the model structure illustrated in **Figure 4**.

**Figure 5:**
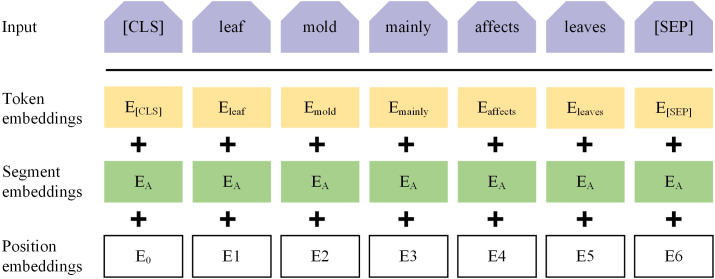
ALBERT input vector representation.

The input representation for ALBERT consists of three embeddings: token embeddings (word embeddings), sentence embeddings, and position embeddings, which are concatenated to clearly represent a text sentence within a token sequence, as shown in **Figure 5**. ALBERT captures both word-level and sentence-level representations through two tasks: Masked Language Model (MLM) and Next Sentence Prediction (NSP), and it is jointly trained on these tasks. In the MLM task, during preprocessing, the target text is first segmented at the character level, and then random MASK operations are applied to the segmented characters. By randomly masking characters in the training text, the Transformer model is forced to build associations within the context of each input character unit, enhancing the ability to capture text features. Additionally, special tokens [CLS] and [SEP] are added at the beginning of the text and between sentences, respectively, to form the final sequence vector for further processing by the Transformer model. For example, in the sentence “*Leaf mold mainly affects leaves*”, if ‘*leaves’* is randomly masked, the resulting sequence vector might be “[CLS] *Leaf mold mainly affects leaves [MASK]*[SEP]”. In the NSP task, the model receives two sentences as input and predicts whether the second sentence is the subsequent sentence of the first one. This task helps the model understand the logical relationship and coherence between sentences.

The Transformer part is the key structure of the ALBERT model, mainly consisting of input, output, and the Encoder section. The Encoder section includes one Self-Attention layer, one Feed Forward layer, and two Add & Norm layers. The Self-Attention layer is the core of the Encoder, receiving the word vector sequence transformed by the embedding layer as input. It generates contextually relevant representations for each word, with the computation method described in Formula 1. In the sentence “*Leaf mold mainly affects leaves*”, the Self-Attention layer balances and integrates the semantic information of each word, producing updated word vector representations such as “‘*leaf mold*’, ‘*mainly’*, ‘*affects’*, ‘*leaves’*”.


(1) $$\text{Attention}\left(Q,K,V\right)=\text{softmax}\left(\frac{QK^{T}}{\sqrt{d_{K}}}\right)V$$

In Formula 1, Q ,  K , and  V are the input word vector matrices, and $\;d_{K}$ is the dimension of the K matrix. The Feed Forward layer performs nonlinear transformations and mappings on the context-aware vectors of each word, such as “leaf mold”, “mainly”, “affects”, and “leaves”, to enhance their feature representation. The Add & Norm layer combines the original input with the processed vectors through residual connections and performs layer normalization to ensure model training stability and accelerate convergence.

#### 2.2.2 BiLSTM module

LSTM, which stands for Long Short-Term Memory, is essentially a type of recurrent neural network (RNN). It is primarily composed of four parts: the input gate, output gate, forget gate, and memory cell (Ma et al., 2021).

The forget gate determines how much of the previous cell state $c_{t-1}$ should be retained in the current cell state $c_{t}$ at time t . The calculation method is given by Formula 2:


(2) $$f_{t}=\sigma (W_{f}•\left[h_{t-1},x_{t}\right]+b_{f})$$

In Formula 2, $f_{t}$ represents the operation of the forget gate at time t , σ is the sigmoid function, $W_{f}$ is the parameter matrix, $h_{t-1}$ is the previous state vector, $x_{t}$ is the input vector at time t , and $\left[h_{t-1},x_{t}\right]$ denotes the concatenation of the two vectors. $b_{f}$ is the bias term. The calculation methods for the input gate are given by Formulas 3–5:


(3) $$i_{t}=\sigma (W_{i}•\left[h_{t-1},x_{t}\right]+b_{i})$$

(4) $$\tilde{c_{t}}=\text{tanh}(W_{c}•\left[h_{t-1},x_{t}\right]+b_{c})$$

(5) $$c_{t}=f_{t}⊗c_{t-1}+i_{t}⊗\tilde{c_{t}}$$

In Formula 3, $i_{t}$ represents the operation of the input gate at time t , which calculates how much of the current input $x_{t}$ should be retained in the cell state $c_{t}$ . Here, σ is the sigmoid function, $W_{i}$ is the parameter matrix, and $b_{i}$ is the bias term. The cell state $c_{t}$ is computed using Formulas 4, 5, where the previous cell state $c_{t-1}$ is element-wise multiplied by the forget gate $f_{t}$ , and the current input cell state $\tilde{c_{t}}$ is element-wise multiplied by the input gate $i_{t}$ , with the two products then summed. The output gate controls the cell state ${\text{c}}_{\text{t}}$ , and its computation is given by Formulas 6, 7:


(6) $$o_{t}=\sigma (W_{o}•\left[h_{t-1},x_{t}\right]+b_{o})$$

(7) $$h_{t}=o_{t}⊗tanhc_{t}$$

In the formulas, $o_{t}$ represents the operation of the output gate at time t , and $h_{t}$ denotes the final cell output value.

**Figure 6:**
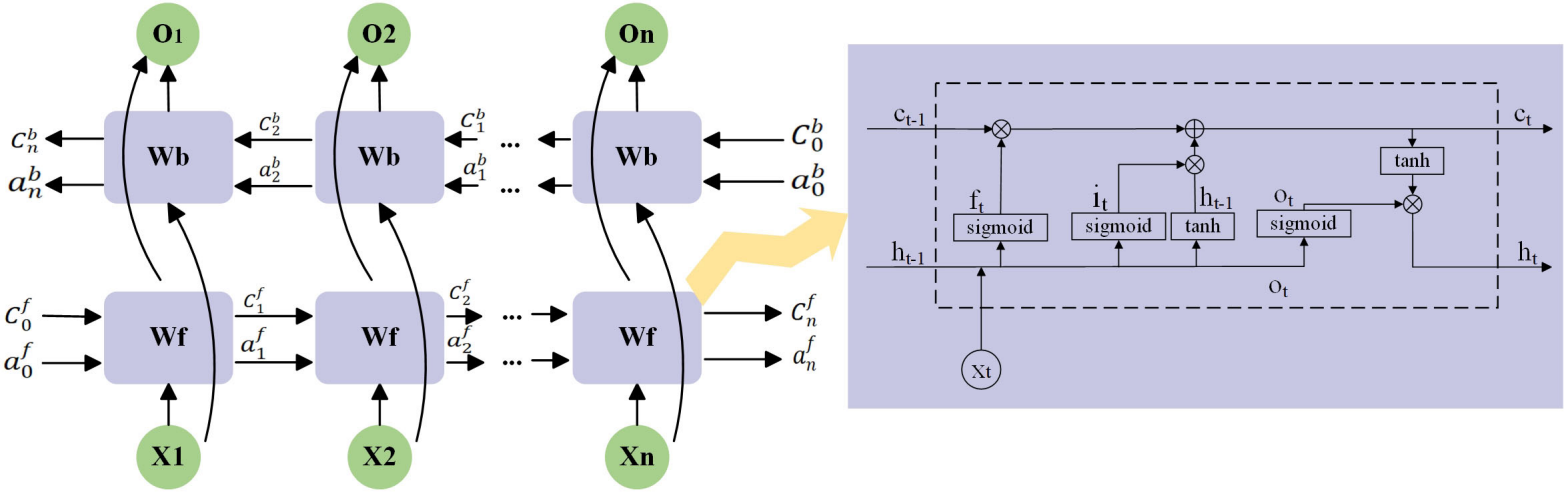
BiLSTM model structure.

Given that unidirectional LSTM networks can only process information in one direction and cannot account for context from both directions, researchers later introduced BiLSTM, or Bidirectional Long Short-Term Memory networks. BiLSTM employs one forward LSTM and one backward LSTM, and merges the outputs from the same time step. This allows the model to capture dependencies between words in the text sequence and the contextual information, providing a more accurate and comprehensive feature representation for tasks such as entity extraction and classification (Lu et al., 2023). The specific structure is shown in **Figure 6**.

#### 2.2.3 CRF module

In the entity extraction process, the CRF module is responsible for optimizing the annotation sequences output by the BiLSTM. It can label disease-related terms in the text data and perform joint modeling of these labels. By constructing a probabilistic model based on a designed score function, the CRF computes probabilities to select the optimal path among possible paths, resulting in accurate entity extraction.

## 3 Results and discussion

### 3.1 Data collection and preprocessing

**Figure 7:**
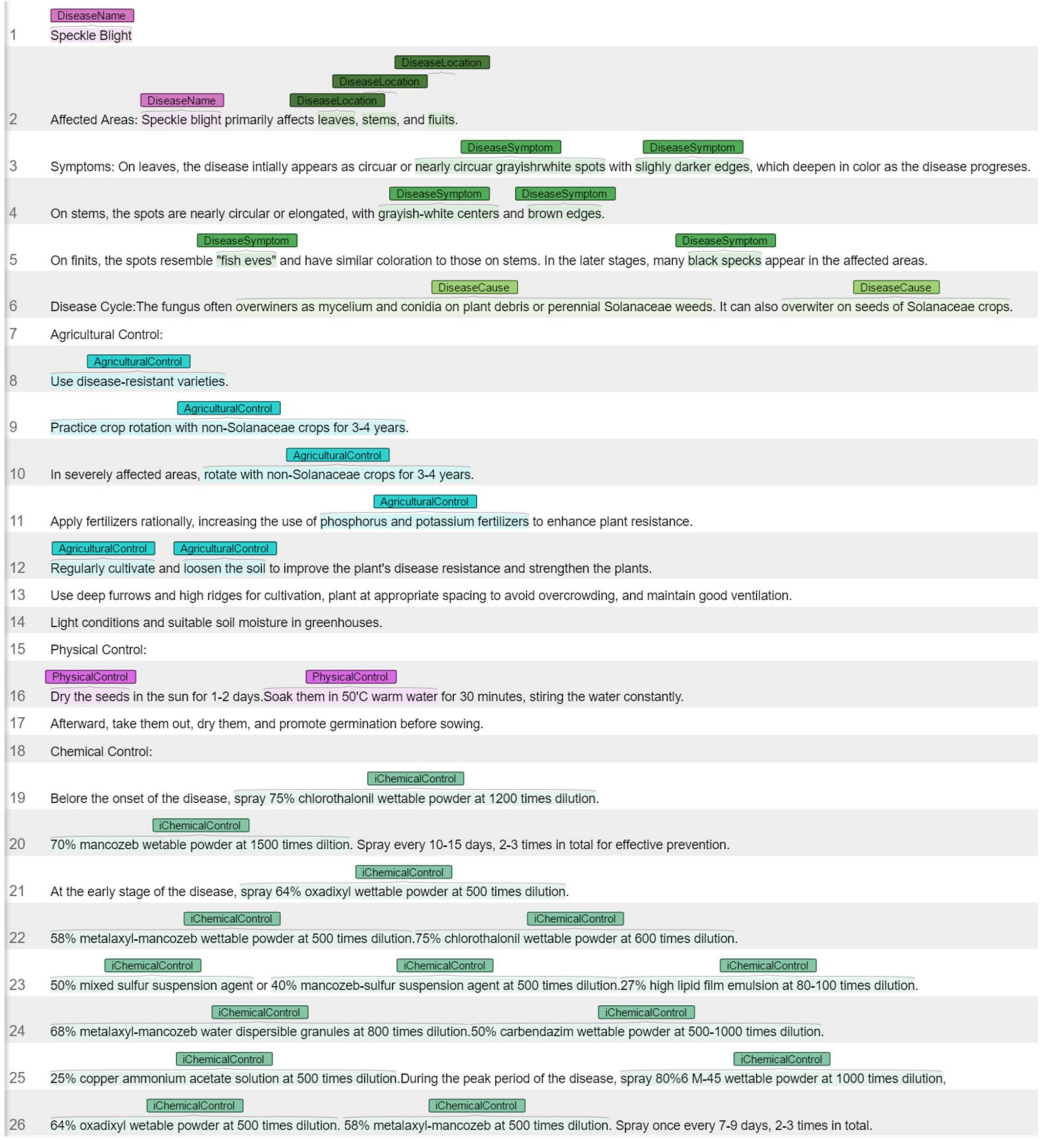
Raw text data on tomato leaf pests and diseases.

Regarding tomato leaf pests and diseases, there is currently no complete and reliable dataset available for knowledge graph construction. This lack of data can impact the accuracy and reliability of analysis models, as the size and diversity of data samples are crucial factors determining model performance. To build a reliable dataset for tomato leaf diseases, we used web scraping techniques to gather a large amount of semi-structured and unstructured text data on tomato pests and diseases from various websites, including: China Agricultural Information Technology Network (https://cast.caas.cn/index.html), National Agricultural Science Data Center (https://www.agridata.cn/#/home), China Pesticide Information Network (http://www.chinapesticide.org.cn/), Baidu Encyclopedia and National Standard Information Public Service Platform (https://std.samr.gov.cn/gb/). Additionally, we scanned electronic books such as Illustrated Guide to Tomato Pest and Disease Diagnosis and Control, Tomato Cultivation and Pest Control Technology Research and New Technologies for Tomato Pest and Disease Control to obtain text data on tomato pests and diseases. The data samples obtained, as shown in **Figure 7**, indicate clear relationships between different entities. Taking speckle blight disease as an example, speckle blight serves as the disease name entity, disease location the entities representing infection sites such as leaves, stems, and fruits. The disease symptoms, such as nearly circular grayish-white spots with slightly darkened edges, grayish-white centers, and brown edges. These symptom entities describe the external characteristics of speckle blight disease. Additionally, the disease cause entity indicates that the disease overwinters through mycelium and conidia on crop residues and perennial solanaceous weeds. In terms of control, there are multiple methods and related entities. Agricultural control entities include the use of disease-resistant varieties, practicing crop rotation with non-Solanaceae for 3-4 years, and applying phosphorus and potassium fertilizers. Physical control entities involve sun-drying seeds and soaking them in warm water. Chemical control entities consist of various pesticides, such as 75% chlorothalonil wettable powder at 1200 times dilution and 64% oxadixyl wettable powder at 500 times dilution, which are used for chemical control of the disease.

Before extracting entities related to tomato leaf pests and diseases, it is necessary to preprocess the dataset. A good preprocessing process ensures the high quality and accuracy of the dataset, facilitating subsequent tokenization and annotation work. With the assistance of domain experts, the preprocessing of the raw sample data includes normalization, elimination of irrelevant information and noise data, and correction of possible language errors to ensure the consistency and reliability of the data. Through systematic preprocessing, we constructed a NER dataset for tomato leaf diseases and pests—the Tomato Leaf Pest and Disease Dataset—providing reliable data support for the construction of a knowledge graph. The dataset includes a training set (1160) and a test set (290), split in an 8:2 ratio.

### 3.2 Evaluation metrics

We set up a training environment suitable for six models: BiLSTM-CRF, 1DCNN-CRF, 1DCNN-CRF-2, BiLSTM-Attention-CRF, BERT-BiLSTM-CRF, and ALBERT-BiLSTM-CRF. To evaluate the performance of the models, we used the same three evaluation metrics commonly found in existing NER papers: Precision (P), Recall (R), and the F1-score as the evaluation standards. The formulas are defined as Equations 8–10, where TEN represents the number of correctly identified entities, FEN is the number of falsely identified non-entities, and NC is the number of correctly identified entities that were not recognized.


(8) $$P=\frac{TEN}{TEN+FEN}\times 100\%$$

(9) $$R=\frac{TEN}{TEN+NC}\times 100\%$$

(10) $$F1=\frac{2\times P\times R}{P+R}\times 100\%$$

### 3.3 Parameter settings

To ensure the reliability of the experimental results, each model was initialized with the same parameters before the study, as shown in **Table 4**. In this table, “Optimizer”refers to the optimization algorithm, “Loss_Function” refers to the loss function, “Learning_Rate”refers to the learning rate, “Lstm_Units” refers to the dimensionality of the output space of the LSTM network, “Batch_Size” refers to the amount of data processed by the model at one time, “Drop_Rate” refers to the dropout rate, “Max_Len” refers to the maximum length of the data processed, and “Epoch” refers to the training cycles of the model.

**Table 4:** Model parameter settings.

Parameter Name	Model Name	Parameter Value
Optimizer	All	Adam
Loss_Function	All	crf.sparse_loss
Learning_Rate	All	1e-5
Lstm_Units	All	128
Batch_Size	ALBERT-BiLSTM-CRF	16
	Others	64
Drop_Rate	ALBERT-BiLSTM-CRF	0.1
	Others	0.5
Max_Len	All	200
Epoch	All	30

### 3.4 Experimental results analysis

The performance of the six models—BiLSTM-CRF, 1DCNN-CRF, 1DCNN-CRF-2, BiLSTM-Attention-CRF, BERT-BiLSTM-CRF, and ALBERT-BiLSTM-CRF—in entity recognition on the Tomato Leaf Disease Dataset is shown in **Table 5**.

**Table 5:** Comparison of entity recognition performance for tomato leaf pests and diseases.

Model Name	Precision/%	Recall/%	F1-score/%
BiLSTM-CRF	87.73	86.31	87.02
1DCNN-CRF	85.39	87.14	86.05
1DCNN-CRF-2	84.92	89.74	87.26
BiLSTM-Attention-CRF	85.15	84.83	85.01
BERT-BiLSTM-CRF	93.10	90.84	91.96
ALBERT-BiLSTM-CRF	95.10	95.03	95.48

**Figure 8:**
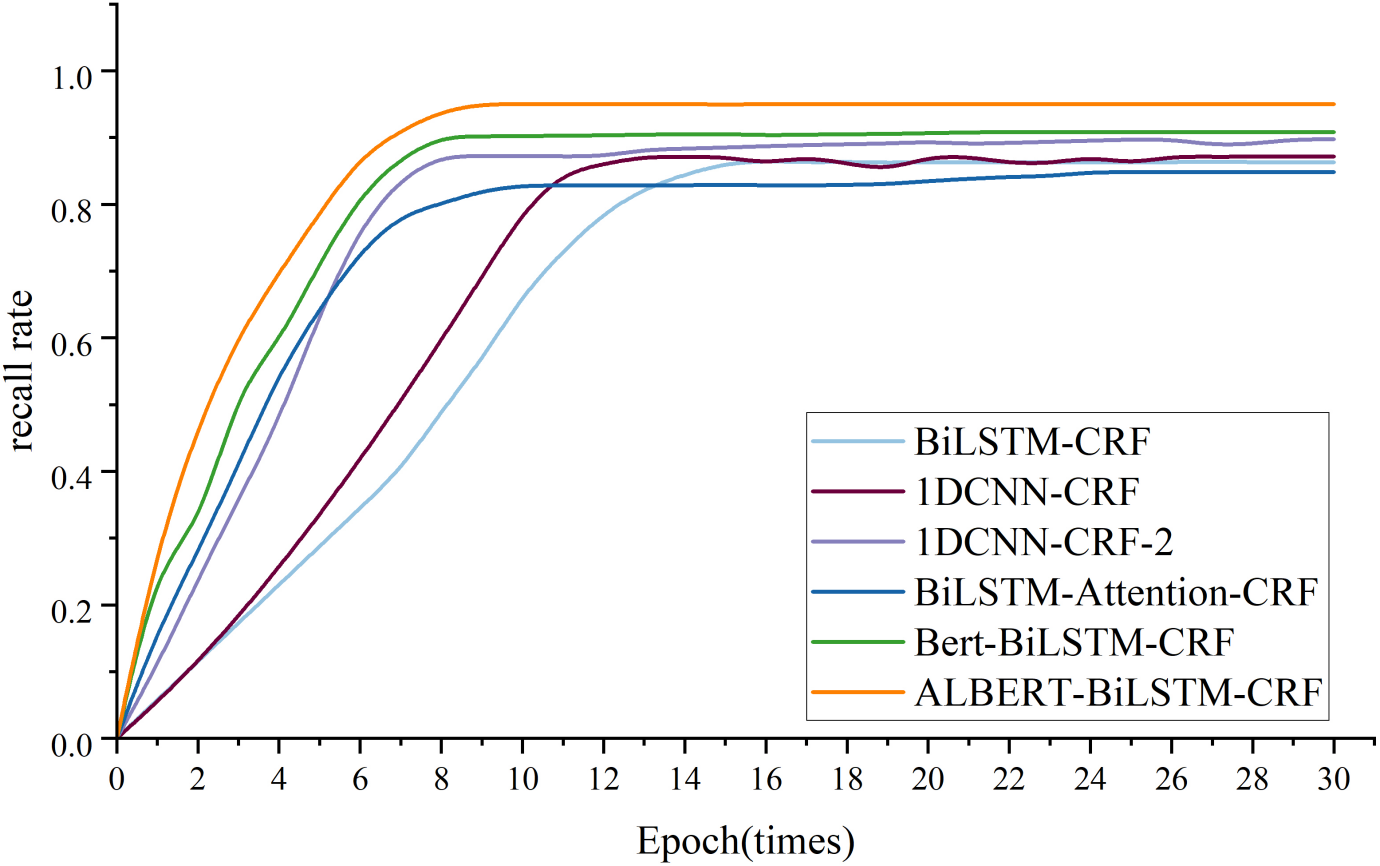
Comparison of model recall rates.

As shown in **Figure 8**, the traditional BiLSTM-CRF and 1DCNN-CRF models have relatively slow convergence speeds, approaching convergence only around the 12th epoch, with a recall rate of approximately 0.85. The BiLSTM-Attention-CRF model converges the fastest but has a lower final recall rate of about 0.80. The ALBERT-BiLSTM-CRF model achieves both fast convergence and the highest recall rate, approximately 0.95. The BERT-BiLSTM-CRF model follows, with a recall rate of around 0.90, while the improved 1DCNN-CRF-2 also has a high recall rate of about 0.89. This indicates that pre-trained models (BERT or ALBERT) significantly enhance model performance and convergence speed compared to the base BiLSTM-CRF model. Additionally, incorporating attention mechanisms or improving CNN structures also contributes to performance improvements. Considering model performance, convergence speed, and complexity, we use the ALBERT-BiLSTM-CRF model for the knowledge extraction task in the subsequent system implementation.

### 3.5 System design and implementation

**Figure 9:**
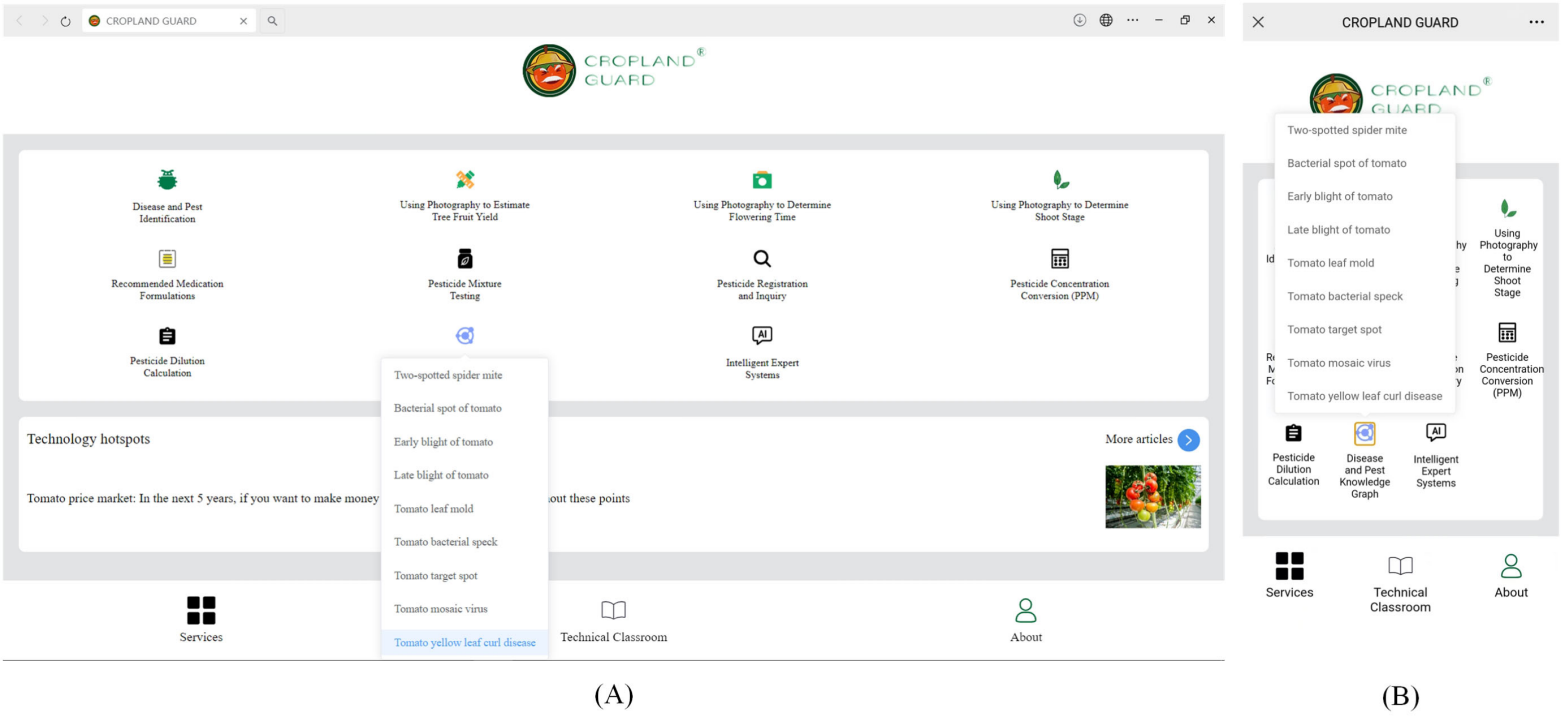
Selecting a specific pest or disease: **(A)** PC, **(B)** Mobile.

The tomato leaf disease and pest knowledge graph system we designed is developed based on the Windows 10 64-bit operating system, using VSCode as the integrated development tool, Vue3 as the frontend development framework, Neo4j as the graph database, and ECharts for knowledge graph visualization. The system supports cross-platform usage (both PC and mobile), ensuring a good user experience across various devices. Users can select a specific pest or disease name from the list and then view the related symptoms visually. As shown in **Figures 9A, B**, when the system receives the query for “*yellow leaf curl disease*”, it first executes the corresponding Cypher query to retrieve information about nodes related to the “*yellow leaf curl disease*” entity.

**Figure 10:**
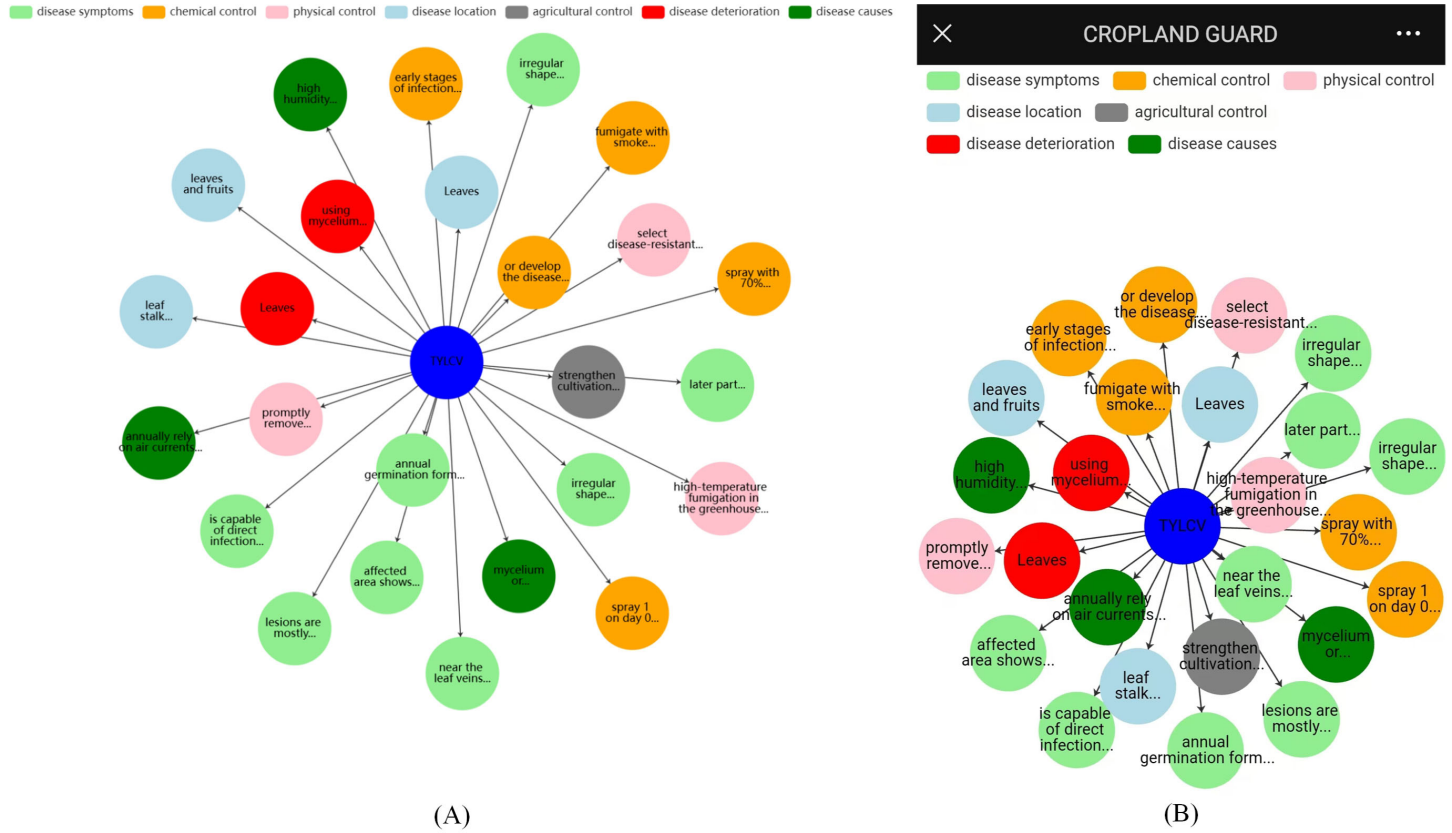
Pest and disease entity query display: **(A)** PC, **(B)** Mobile.

ECharts is then used to render the entity and node information, and the results are finally displayed to the user in **Figures 10A, B**.

## 4 Dicussion

For the tomato leaf pest and disease domain, we collected and organized relevant data to construct a dataset, TLP2D, containing approximately 10,400 entities and 1,450 samples. Compared to other corpora in the same field, our dataset not only includes a broader range of categories, but also offers more detailed descriptions, providing timely decision analysis for tomato leaf pest and disease control. By comparing different entity extraction models, we found that the ALBERT-BiLSTM-CRF model performs well on TLP2D. This model leverages the strengths of ALBERT, BiLSTM, and CRF models to effectively extract entities from TLP2D, supporting the construction of a tomato leaf pest and disease knowledge graph and providing a reference for knowledge graph construction in other fields. The development of the tomato leaf pest and disease digital diagnostic system offers precise pest and disease control recommendations for farmers, lowers the barrier to professional knowledge sharing, and introduces new ideas for pest and disease management. However, we also recognize that there is room for improvement in this work. Future efforts will focus on expanding the domain scope, optimizing data annotation methods, and continuously fine-tuning the NER model to better serve agriculture.

## Data Availability

The raw data supporting the conclusions of this article will be made available by the authors, without undue reservation.
